# Accurate fully automated assessment of left ventricle, left atrium, and left atrial appendage function from computed tomography using deep learning

**DOI:** 10.1093/ehjimp/qyaf011

**Published:** 2025-03-06

**Authors:** Lee Jollans, Mariana Bustamante, Lilian Henriksson, Anders Persson, Tino Ebbers

**Affiliations:** Center for Medical Image Science and Visualization, Linköping University, SE-581 83 Linköping, Sweden; Department of Health, Medicine, and Caring Sciences, Linköping University, SE-581 83 Linköping, Sweden; Center for Medical Image Science and Visualization, Linköping University, SE-581 83 Linköping, Sweden; deCODE Genetics/Amgen Inc., Sturlugata 8, 101 Reykjavik, Iceland; Center for Medical Image Science and Visualization, Linköping University, SE-581 83 Linköping, Sweden; Department of Health, Medicine, and Caring Sciences, Linköping University, SE-581 83 Linköping, Sweden; Department of Radiology, Linköping University, SE-581 83 Linköping, Sweden; Center for Medical Image Science and Visualization, Linköping University, SE-581 83 Linköping, Sweden; Department of Health, Medicine, and Caring Sciences, Linköping University, SE-581 83 Linköping, Sweden; Department of Radiology, Linköping University, SE-581 83 Linköping, Sweden; Center for Medical Image Science and Visualization, Linköping University, SE-581 83 Linköping, Sweden; Department of Health, Medicine, and Caring Sciences, Linköping University, SE-581 83 Linköping, Sweden

**Keywords:** deep learning, left ventricular ejection fraction, stroke volume, cardiac segmentation, computed tomography, vision transformer

## Abstract

**Aims:**

Assessment of cardiac function is essential for diagnosis and treatment planning in cardiovascular disease. Volume of cardiac regions and the derived measures of stroke volume (SV) and ejection fraction (EF) are most accurately calculated from imaging. This study aims to develop a fully automatic deep learning approach for calculation of cardiac function from computed tomography (CT).

**Methods and results:**

Time-resolved CT data sets from 39 patients were used to train segmentation models for the left side of the heart including the left ventricle (LV), left atrium (LA), and left atrial appendage (LAA). We compared nnU-Net, 3D TransUNet, and UNETR. Dice Similarity Scores (DSS) were similar between nnU-Net (average DSS = 0.91) and 3D TransUNet (DSS = 0.89) while UNETR performed less well (DSS = 0.69). Intra-class correlation analysis showed nnU-Net and 3D TransUNet both accurately estimated LVSV (ICC_nnU-Net_ = 0.95; ICC_3DTransUNet_ = 0.94), LVEF (ICC_nnU-Net_ = 1.00; ICC_3DTransUNet_ = 1.00), LASV (ICC_nnU-Net_ = 0.91; ICC_3DTransUNet_ = 0.80), LAEF (ICC_nnU-Net_ = 0.95; ICC_3DTransUNet_ = 0.81), and LAASV (ICC_nnU-Net_ = 0.79; ICC_3DTransUNet_ = 0.81). Only nnU-Net significantly predicted LAAEF (ICC_nnU-Net_ = 0.68). UNETR was not able to accurately estimate cardiac function. Time to convergence during training and time needed for inference were both faster for 3D TransUNet than for nnU-Net.

**Conclusion:**

nnU-Net outperformed two different vision transformer architectures for the segmentation and calculation of function parameters for the LV, LA, and LAA. Fully automatic calculation of cardiac function parameters from CT using deep learning is fast and reliable.

## Graphical Abstract

**Graphical Abstract:**
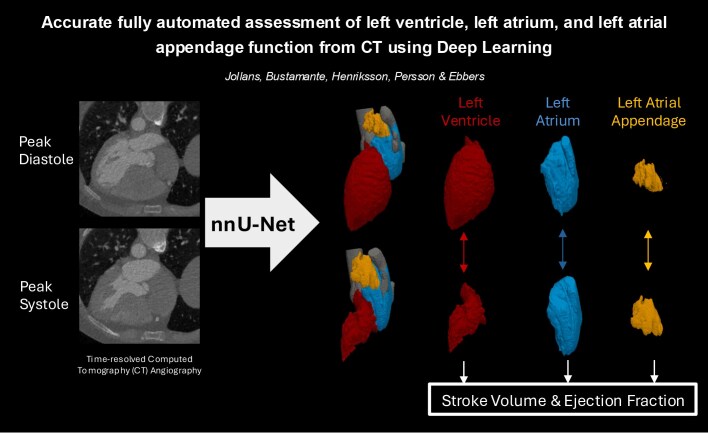

## Introduction

Cardiac remodelling is an important indicator of future pathology and mortality. Higher left atrium (LA) volume is associated with symptom development, heart failure hospitalizations, aborted cardiac arrest, and cardiovascular death.^1–3^ Low left ventricular (LV) ejection fraction (EF) is associated with higher rates of hospitalizations and mortality, while high LVEF after myocardial infarction is associated with reduced mortality.^4–8^ The volume and shape of the left atrial appendage (LAA) are also important indicators for risk of onset and recurrence of atrial fibrillation, which in turn greatly increases the risk for stroke.^9–11^ Due to the association of cardiac structure and function with cardiovascular pathology risk, efficient methods of monitoring cardiac remodelling are of great clinical importance.^12^ Attempts to use deep learning (DL) to automate the segmentation of cardiac structures have shown that fully automated methods are of similar or superior quality compared with manual segmentations, while requiring substantially less time, and being more reproducible.^13–15^ The majority of work using fully automatic methods to process cardiovascular imaging data has focussed on segmentation without assessment of cardiac function parameters.^16^ However, a number of previous studies have used DL to predict EF of the right or LV from echocardiography^17–19^ or magnetic resonance imaging (MRI).^20,21^

Echocardiography is the standard clinical tool for assessment of cardiac function^22^ and has the advantages of high temporal resolution and low cost. However, methods for volume calculation from echocardiography (e.g. biplane disk summation) rely on assumptions about the shape of the ventricle.^23^ Measurements of LVEF can vary substantially between raters^24^ and have been shown to agree best between echocardiography and computed tomography (CT) and between CT and MRI.^25,26^ Compared with MRI, CT can offer higher spatial resolution and is therefore preferable for precise anatomical delineation of cardiac structures.^27,28^ Since CT is often part of clinical routine for patients with heart disease,^22^ a fully automatic method of assessing important metrics of cardiac function from CT would be of great benefit.

Methods for segmenting the LA and LV from CT using DL achieve high accuracy^29–31^ and have been reported to correlate with invasive haemodynamics measurements.^14^ Automatic segmentation of the LAA remains less studied and more challenging since the shape of the LAA varies widely between patients.^32^ Furthermore, contrast media may not fully penetrate the LAA due to high blood residence time and low blood flow velocity in parts of the LAA farther from the LAA orifice.^33,34^ A small number of studies have developed automatic segmentation methods for the LAA from CT, with the goal of use in clinical routine,^35^ identification of LAA filling defects,^36^ or preparation for occluder placement.^37^

The most commonly used DL method for medical image segmentation is the U-Net, with great success achieved by the self-configuring implementation nnU-Net.^38^ U-Net is a convolutional neural network, which, constrained by kernel size, typically only evaluates small patches of voxels at once, limiting its field of view. Recently, a great deal of attention has been given to vision transformers (ViT)^39^ as a way to counteract this limitation by enabling long-range spatial dependencies. Proposed network architectures including ViT for image processing include TransUNet^40^ and UNETR.^41^

In this study, we evaluated the agreement between cardiac function parameters based on manual segmentation of time-resolved CT angiography and estimations of cardiac function parameters [end-diastolic volume (EDV), end-systolic volume (ESV), stroke volume (SV), and EF] from 3D DL methods for the LV, LA, and LAA. The self-configuring U-Net implementation nnU-Net was compared with two different DL architectures incorporating ViT blocks: UNETR and TransUNet.

## Methods

### Study data set

A total of 767 image volumes were used for training, validation, and testing. The image volumes represented time-resolved CT data sets from 39 patients, which were part of a larger sample of cardiac CT data from routine medical electrocardiogram-gated coronary CT angiography examinations approved for retrospective research use. All data sets were acquired on a dual source 192 ∗ 192 slice CT scanner (Siemens Somatom Force, Siemens, Forchheim, Germany). Twenty time instances representing the whole cardiac cycle were acquired for each patient. Data were acquired at 512 ∗ 512 voxel resolution with between 373 and 561 slices with 0.5 mm thickness and 0.25 mm overlap. Pixel size varied between 0.27 and 0.41 mm (mean: 0.33; standard deviation: 0.034). For one patient, all data were excluded, and for a further 13, volumes were excluded due to poor image quality. The data underlying this article cannot be shared publicly due to the terms of the ethical approval allowing their use in this research. If possible under the ethical approval, the data will be shared on reasonable request to the corresponding author.

### Ground truth generation

**Figure 1:**
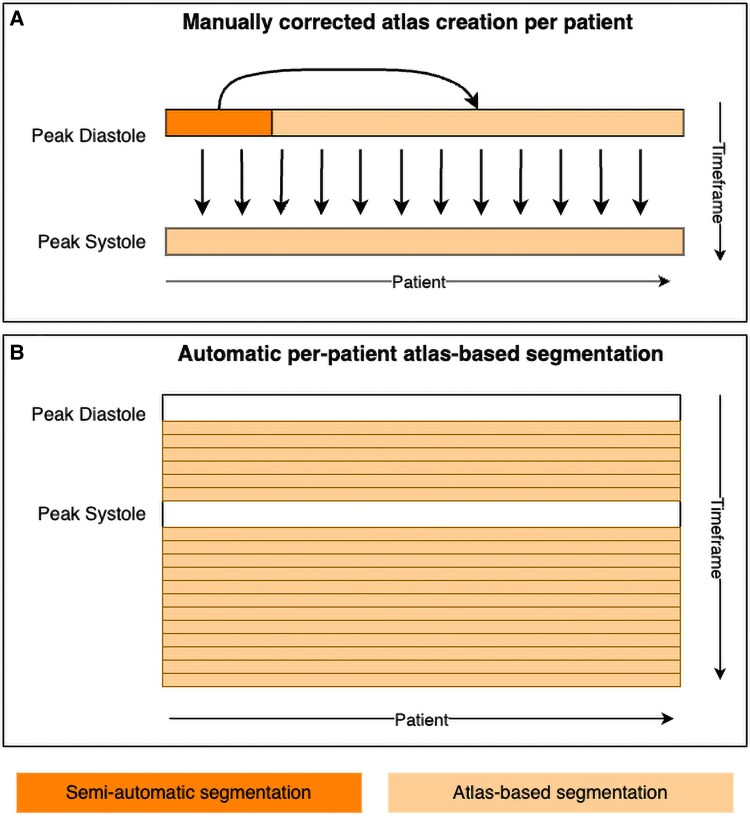
Ground truth creation schematic. (*A*) Semi-automatic segmentation of the peak-diastolic timeframe in ITK-Snap using the ‘active contour’ method for a subset of patients (*n* = 8) followed by atlas-based segmentation of the remaining peak-diastolic volumes (*n* = 31). The peak-diastolic timeframes were used for atlas-based segmentation of the peak-systolic timeframes in each patient separately. All segmentations for the peak-diastolic and peak-systolic timeframe were manually corrected. (*B*) Automatic atlas-based segmentation for the remaining timeframes based on the previously segmented peak-systolic and peak-diastolic timeframe for each patient separately.

The ground truth was generated using semi-automatic segmentation in ITK-Snap^42^ using the ‘active contour’ method and atlas-based segmentation. Multi-atlas-based segmentations were obtained using the approach described in Bustamante *et al*.^43^ Hereby, atlas is used to refer to a previously labelled image. Using registration, a transformation between a different target image and the atlas is estimated and the labels of the atlas are propagated into the target’s image space. For multi-atlas segmentation, the transformed labels from multiple atlases are combined into one segmentation. The LA, LV, ascending aorta (AA), pulmonary veins (PVs), and LAA were segmented. Only the end-systolic and the end-diastolic timeframe were manually corrected and used to create atlases to automatically segment the remaining time instances. These time instances were selected to ensure that the most extreme changes in cardiac volume were correctly depicted. The process for ground truth creation is shown in *Figure 1*. Visual inspection spot checks of the automatically generated segmentations were carried out for each patient. This resulted in segmentations for two patients being manually corrected for two time instances each.

### DL architectures

The ‘3D low-res’ setting was used in nnU-Net.^38^ For UNETR, the implementation in the MONAI DL package for medical imaging^44^ was used. TransUNet, which was written for 2D inputs, has a publicly available implementation which we modified for 3D inputs. The code for our implementation of this network is available at github.com/ljollans/TRUNet.

### Network training

Analyses were conducted using PyTorch 1.9^45^ on a NVIDIA DGX A100 80GB GPU. The GPU memory limited the possible batch size to 2. Data were split into training (*N* = 560, 28 patients) and test set (*N* = 207, 11 patients) at the patient level prior to any analyses being carried out to keep these splits consistent across different methods. For UNETR and 3D TransUNet, a split into training (*N* = 440, 22 patients) and validation set (*N* = 120, 6 patients) was defined a priori. nnU-Net automatically implements a five-fold cross-validation. nnU-Net, UNETR, and 3D TransUNet were trained to create segmentation masks for the LA, LV, LAA, AA, and PV based on single 3D image volumes without information about time instance. For UNETR and 3D TransUNet, the learning rate was initialized at 0.01, decaying with each iteration by power 0.9 using the poly learning rate strategy. The loss function used was the mean of dice loss and cross-entropy loss. The background was not included in the calculation of the loss function. Adam Optimizer was used for training.^46^ UNETR and 3D TransUNet were trained for 72 h, regardless of the number of iterations and epochs completed. This constraint was chosen based on limitations imposed by the computing architecture used in this study but was judged as appropriate based on convergence and comparison to the results achieved by nnU-Net. The model that resulted in the highest Dice Similarity Score (DSS) in the validation set was selected and applied to the test set. Segmentation performance was evaluated quantitatively using the DSS.

### Data preprocessing

For 3D TransUNet and UNETR, volumes were downsampled to be 224 pixels large along every axis, in line with previous work using ViT.^39,40^ In each epoch, the data were randomly rotated by up to 20° along a randomly selected axis with a likelihood of 50%, and randomly flipped along a randomly selected axis with a likelihood of 50%.

### Data postprocessing

Only the largest connected component was retained in the segmentation for each region of interest (ROI) except the PV. For the PV, all clusters that were at least half as large (in terms of voxel extent) as the largest PV component were retained. The postprocessing automatically selected by nnU-Net also retained the largest component for all ROIs except the PV.

For patients in the test set, for whom all 20 time instances were available (*N* = 10), the end-diastolic and end-systolic time instances were identified by the maximum and minimum LV volumes, respectively. This was done separately for each method of volume calculation in order to evaluate performance of each method in isolation. For the LV, LA, and LAA, the EDV, ESV, SV, and EF were calculated.

### Statistical analysis

EDV, ESV, SV, and EF were compared between the ground truth and the DL methods. Normality was assessed using the Shapiro–Wilk test. Since most measurements did not meet the assumption of normality, Wilcoxon signed-rank tests were carried out. Agreement between the ground truth and the DL methods was assessed through Bland–Altman analysis and intra-class correlation coefficient. Correction for multiple comparisons was done using the Benjamini–Hochberg method.

## Results

The highest dice score for all ROIs was achieved by nnU-Net, closely followed by 3D TransUNet. UNETR produced markedly lower dice scores for all ROIs (*Table 1*).

**Table 1:** Quantitative comparison of segmentation performance: DSS

	LV	LA	LAA	AA	PV
nnU-Net	0.9564	0.9606	0.8534	0.9754	0.8039
3D TransUNet	0.9511	0.9507	0.8102	0.9684	0.7694
UNETR	0.8094	0.6621	0.6529	0.6760	0.6406

AA, ascending aorta; LA, left atrium; LAA, left atrial appendage; LV, left ventricle; PV, pulmonary veins.

**Figure 2:**
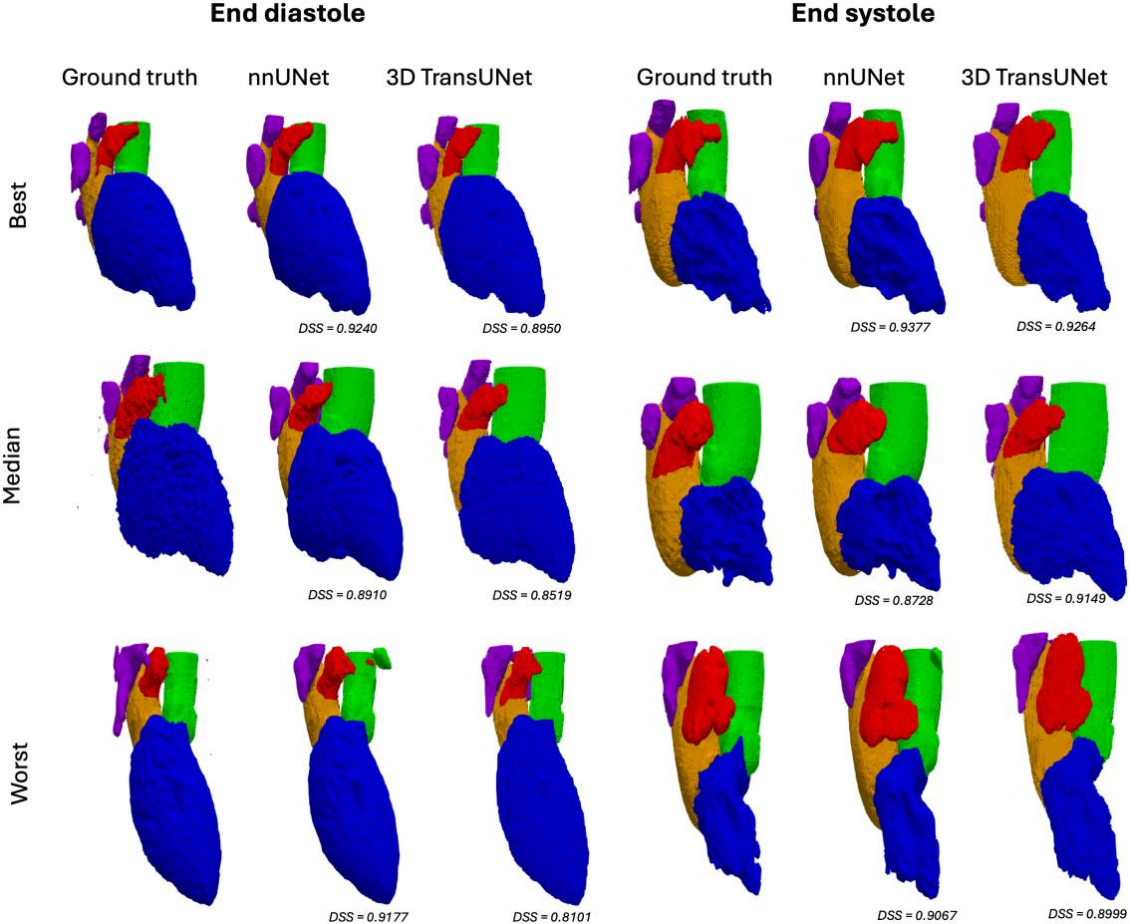
Rendering of segmentation result from three representative patients (rows) from the test set at peak diastole and peak systole. The patients were selected based on average DSS across time points. The average DSS for ‘best’ is 0.9392 for nnU-Net and 0.9226 for 3D TransUNet. The average DSS for ‘median’ is 0.9132 for nnU-Net and 0.8988 for 3D TransUNet. The average DSS for ‘worst’ is 0.8826 for nnU-Net and 0.8713 for 3D TransUNet. Blue: LV; orange: LA; red: LAA; green: AA; purple: PVs.

**Figure 3:**
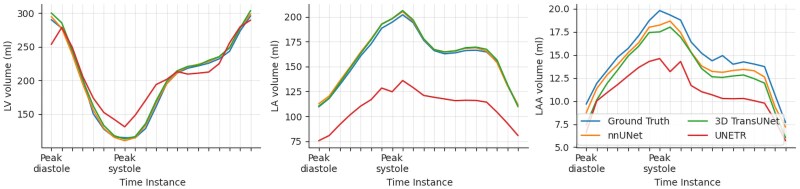
Regional volume across the cardiac cycle. Average volume for patients in the test set calculated for the LV, LA, and LAA across the cardiac cycle. Volumes for all regions are calculated based on the largest connected component for that region in the segmentation.

Based on ICC and Wilcoxon signed-rank tests, the nnU-Net predictions were in line with the ground truth for all measures (*Table 2*). The 3D TransUNet predictions of LAAEF did not show a significant ICC with the ground truth. The UNETR predictions were only in line with the ground truth for EDV and ESV of the LV and LA. *Figure 2* shows 3D renderings of representative cases for the two best-performing methods. *Figure 3* shows estimated LA, LV, and LAA volume across the cardiac cycle for all methods, showing that UNETR consistently underestimated LA and LAA volume.

**Table 2:** Bland–Altman statistics, intra-class correlation analysis, and Wilcoxon signed-rank test results for all measures between the ground truth and nnU-Net, 3D TransUNet, and UNETR

			Bland–Altman analysis		Correlation analysis			Wilcoxon signed-rank test	
			Mean bias (%)	Limits of agreement (%)	Intra-class correlation coefficient	95% confidence interval	*P*-value	*W*	*P*-value
Left ventricle	EDV	nnU-Net	2.58	−4.76 to 9.93	1.00	0.98–1.00	<0.0001^*^	22	0.62500
		3D TransUNet	0.45	−4.38 to 5.27	0.99	0.96–1.00	<0.0001^*^	10	0.08398
		UNETR	0.08	−23.48 to 23.65	0.92	0.72–0.98	<0.0001^*^	25	0.84570
	ESV	nnU-Net	1.54	−7.29 to 10.36	1.00	1.00–1.00	<0.0001^*^	13	0.16016
		3D TransUNet	−0.94	−7.34 to 5.46	1.00	0.99–1.00	<0.0001^*^	24	0.76953
		UNETR	12.61	−85.50 to 110.73	0.89	0.63–0.97	0.00011^*^	19	0.43164
	SV	nnU-Net	3.32	−5.19 to 11.84	0.95	0.82–0.99	<0.0001^*^	15	0.23242
		3D TransUNet	1.91	−6.34 to 10.16	0.94	0.77–0.98	<0.0001^*^	8	0.04883
		UNETR	−3.24	−52.86 to 46.38	0.31	−0.35 to 0.77	0.17292	21	0.55664
	EF	nnU-Net	0.72	−3.29 to 4.73	1.00	0.98–1.00	<0.0001^*^	12	0.13086
		3D TransUNet	1.44	−4.06 to 6.94	1.00	0.98–1.00	<0.0001^*^	18	0.37500
		UNETR	−3.99	−43.30 to 35.32	0.59	−0.02 to 0.88	0.02774	16	0.27539
Left atrium	EDV	nnU-Net	−3.21	−16.23 to 9.82	0.99	0.96–1.00	<0.0001^*^	27	1.00000
		3D TransUNet	0.21	−8.17 to 8.59	0.97	0.89–0.99	<0.0001^*^	19	0.43164
		UNETR	−30.28	−58.77 to −1.79	0.92	0.73–0.98	<0.0001^*^	0	0.00195^*^
	ESV	nnU-Net	0.49	−5.43 to 6.41	0.99	0.96–1.00	<0.0001^*^	19	0.43164
		3D TransUNet	0.90	−5.53 to 7.32	0.99	0.95–1.00	<0.0001^*^	20	0.49219
		UNETR	−42.39	−94.67 to 9.90	0.68	0.13–0.91	0.01101^*^	0	0.00195^*^
	SV	nnU-Net	5.81	−13.50 to 25.13	0.91	0.67–0.98	<0.0001^*^	19	0.43164
		3D TransUNet	2.06	−9.88 to 14.00	0.80	0.39–0.95	0.00148^*^	13	0.16016
		UNETR	−53.93	−156.20 to 48.33	0.10	−0.53 to 0.66	0.38088	1	0.00391^*^
	EF	nnU-Net	5.26	−12.24 to 22.77	0.95	0.82–0.99	<0.0001^*^	22	0.62500
		3D TransUNet	1.12	−7.14 to 9.38	0.81	0.41–0.95	0.00115^*^	12	0.13086
		UNETR	−734.74	−4951.50 to 3482.02	−0.00	−0.60 to 0.60	0.50201	16	0.27539
Left atrial appendage	EDV	nnU-Net	−22.59	−75.55 to 30.36	0.81	0.37–0.95	0.00213^*^	14	0.35938
		3D TransUNet	−5.81	−59.73 to 48.11	0.82	0.40–0.96	0.00172^*^	7	0.07422
		UNETR^a^	−4.19	−73.46 to 65.08	0.53	−0.21 to 0.88	0.07217	8	0.09766
	ESV	nnU-Net	−6.42	−31.22 to 18.39	0.90	0.61–0.98	0.00022^*^	12	0.25000
		3D TransUNet	−4.91	−32.58 to 22.76	0.88	0.55–0.97	0.00045^*^	10	0.16406
		UNETR^a^	−21.02	−62.45 to 20.41	0.56	−0.17 to 0.89	0.05956	1	0.01562^*^
	SV	nnU-Net	15.10	−63.42 to 93.62	0.79	0.32–0.95	0.00314^*^	18	0.65234
		3D TransUNet	2.98	−44.94 to 50.89	0.81	0.36–0.95	0.00238^*^	20	0.82031
		UNETR^a^	−24.18	−126.72 to 78.36	0.41	−0.36 to 0.84	0.13828	1	0.01562^*^
	EF	nnU-Net	22.07	−44.05 to 88.20	0.68	0.08–0.92	0.01580^*^	15	0.42578
		3D TransUNet	8.62	−35.56 to 52.79	0.52	−0.17 to 0.87	0.06293	7	0.07422
		UNETR^a^	−9.46	−74.44 to 55.52	0.31	−0.45 to 0.81	0.20645	8	0.19531

EDV, end-diastolic volume; EF, ejection fraction; ESV, end-systolic volume; LA, left atrium; LAA, left atrial appendage; LV, left ventricle; SV, stroke volume.

^a^One patient excluded from analyses due to failure to segment the LAA at peak diastole.

^*^Significant *P*-value according to Benjamini–Hochberg correction.

**Figure 4:**
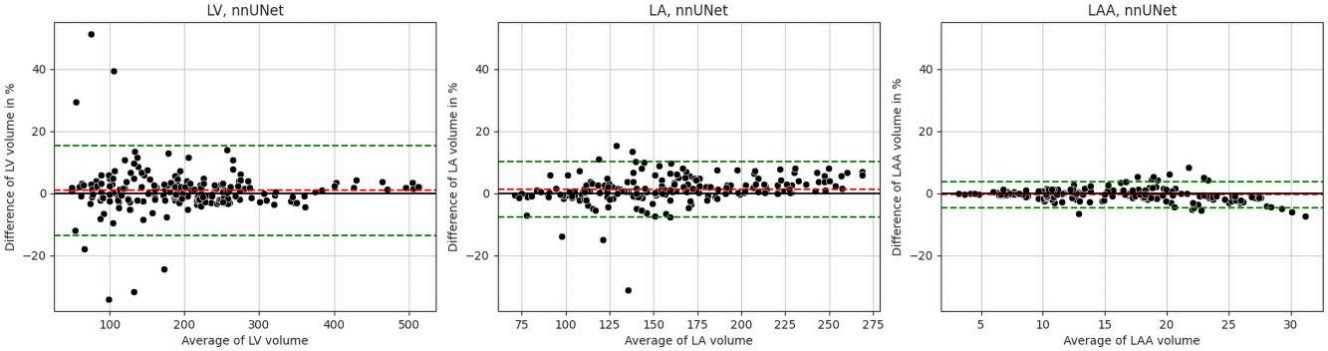
Bland–Altman plots for region volumes calculated using the ground truth and nnU-Net predictions for the LV, LA, and LAA. Plots contain bias (red dashed line) and limits of agreement (±1.96 SD, green dashed lines).

**Figure 5:**
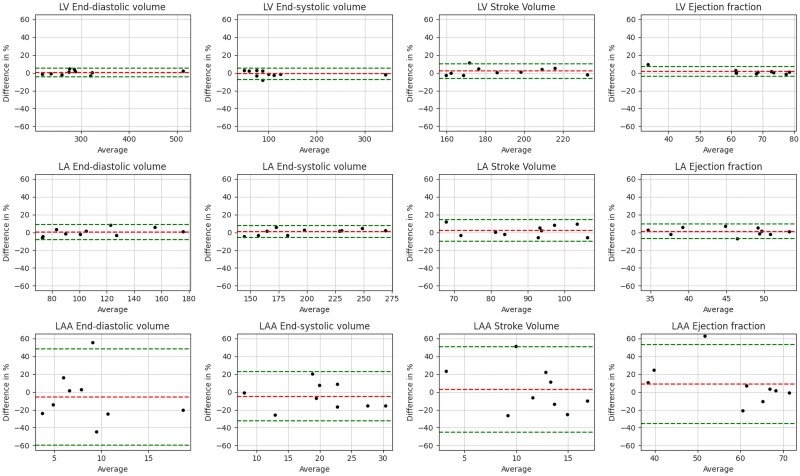
Bland–Altman plots for cardiac function parameters calculated using the ground truth and nnU-Net predictions. Plots contain bias (red dashed line) and limits of agreement (±1.96 SD, green dashed lines).

Bland–Altman statistics showed that the nnU-Net and 3D TransUNet predictions had little bias for estimation of LV and LA functional metrics, but EDV and ESV for the LAA were consistently underestimated and SV and EF overestimated by both methods. Bland–Altman plots for nnU-Net volume estimations and cardiac function parameter estimations are shown in *Figures 4* and *5*, respectively.

Notably, both training and inference times were slower for nnU-Net than for 3D TransUNet. nnU-Net completed training within ∼48 h while 3D TransUNet reached the highest validation set dice score within ∼36 h. Inference time including postprocessing was on average 275.84 s for nnU-Net (minimum: 167.87; maximum: 339.35) while inference time for 3D TransUNet was on average 40.67 s (minimum: 35.97; maximum: 45.15).

## Discussion

In this study, we evaluated fully automatic DL approaches for assessment of cardiac function from time-resolved CT angiography. nnU-Net was able to accurately predict LV, LA, and LAA function, while 3D TransUNet failed to predict EF of the LAA, and UNETR was only able to predict the volume but not the SV or EF of any ROI. This is the first study to show that SV and EF of the LA, LV, and LAA can simultaneously be estimated from CT using a fully automatic 3D DL method.

Of the three DL architectures evaluated here, only nnU-Net^38^ and 3D TransUNet^40^ were able to estimate cardiac function. Both networks use sequential cascade encoders and decoders with skip connections. The skip connections from different parts of the encoder cascade allow the network to derive low-level semantic features and combine them with high-level localization information. In contrast, UNETR^41^ replaces the encoder cascade with a transformer block from which sequence representations are extracted and reshaped before being passed as skip connections to the cascade decoder. These skip connections in the UNETR architecture do not represent different encoding stages or spatial scales. Our findings are in line with conclusions drawn by Roy and several of the authors of nnU-Net that convolutional or Swin operations rather than the transformer block are likely often the reason for the success of ViT architectures.^47,48^ Although 3D TransUNet enabled faster training and inference, our findings confirm that the well-tested automatic configuration implemented in the nnU-Net method remains a good choice for accurate simultaneous multi-region segmentation from medical images.

Several studies have used DL to automate calculation of LVEF from echocardiography^17–19^ and from MRI.^20,21^ Our findings show that DL can also be used to correct and supplement these measurements without substantial time investment or further examinations when CT is acquired as part of routine diagnostic procedure. While studies seeking to automate cardiac segmentations from CT usually include the LA and LV, segmentation of the LAA is more challenging and less well studied. The LAA is typically not included as a ROI in automated segmentations of the heart, being either explicitly excluded^14,49^ or included as part of the LA.^29,37^ In this study, the nnU-Net segmentation achieved a dice score of 0.85 for the LAA, compared with 0.96 for the LA. Conversely, visual examination of the segmentations showed that the consistent undersegmentation of the LAA was typically due to parts of the LAA far from the orifice being missed. This is consistent with findings from computational fluid dynamics (CFD) studies showing a tendency for LAA blood flow to stagnate further from the orifice,^33,34^ which hampers the transport of contrast agent to these areas. A previous study also segmenting the LAA from CT sought to identify filling defects through analysis of Hounsfield number differences.^36^ While the authors report a high dice score of 0.94, this result was based on slices specifically selected for optimal view of the LAA and is therefore not directly comparable with our fully 3D segmentation. Other approaches reported dice scores up to 0.95 but required manual localization of the LAA.^35,50^

Large size and low EF of the LAA are risk factors for onset of atrial fibrillation and for recurrence after ablation.^9,10^ Calculation of these variables in standard clinical practice could improve stratification of patient risk for atrial fibrillation. In this study, we demonstrated that fully automatic estimation of LAAEF from time-resolved CT is feasible. Patient-specific CFD studies have shown that LAAEF is associated with residence time of blood in the LAA,^51^ highlighting the relevance of LAAEF as a potential marker of thrombus risk. In patients with high risk for blood clots, the planning of LAA occlusion also requires accurate segmentation of the LA and LAA. In addition, identification of landmarks such as the mitral valve annulus and the LAA ostium is necessary for presurgical planning. Future work building on DL approaches to identify these landmarks^37^ could automatically create more complete models of cardiac geometry, facilitating the creation of personalized CFD simulations.

### Limitations

While the data set used in this study is large compared with other work training DL models for 3D medical image segmentation, the effective size of the test set on which performance of the different approaches was evaluated is only 10 patients. Furthermore, since these data were completely anonymized prior to this work being carried out, conclusions based on patient characteristics are impossible. Future work examining the reliability of automatic DL calculations of cardiac function markers in different pathophysiological phenotypes and comparing them to measurements from echocardiography will be worthwhile. A limitation of this work regarding the comparison of different network architectures is that training time was capped independently of convergence. For UNETR, segmentation performance in the validation set still showed a very small upward trend at the end of training, indicating that increased training time may have modestly improved performance. However, the discrepancy between segmentation performance for UNETR and the other methods was so large that continued training was judged not to be worthwhile. Since the number of epochs completed by 3D TransUNet and by UNETR within the given timeframe was similar (190 and 198, respectively), the training cut-off based on wall time rather than number of epochs is unlikely to have unfairly disadvantaged one of the methods. Finally, in this study, the impact of the network architecture cannot be examined separately from the extensive preprocessing operations or cross-validation implemented by nnU-Net. Therefore, both 3D TransUNet and UNETR may have benefitted from similar preprocessing steps and from inference being based on multiple cross-validation folds.

## Conclusion

In this study, we show that fully automatic calculation of LV, LA, and LAA volume and function from CT is feasible using DL. For the segmentation task and subsequent calculation of cardiac function parameters, the self-configuring nnU-Net architecture outperformed two different ViT architectures.

## Lead author biography

Lee Jollans received their PhD from the Faculty of Psychology at Trinity College Dublin in 2019 using supervised machine learning to investigate MRI and EEG data associated with substance use. Subsequently, they studied the variation in brain structure between patient groups with affective disorders at the Max Planck Institute for Psychiatry in Munich using unsupervised learning. Since 2023, Lee has shifted their research focus to cardiac imaging and is developing deep learning models in clinical cardiology at Linköping University.
